# Amorphous nickel boride membrane on a platinum–nickel alloy surface for enhanced oxygen reduction reaction

**DOI:** 10.1038/ncomms12362

**Published:** 2016-08-09

**Authors:** Daping He, Libo Zhang, Dongsheng He, Gang Zhou, Yue Lin, Zhaoxiang Deng, Xun Hong, Yuen Wu, Chen Chen, Yadong Li

**Affiliations:** 1 Center of Advanced Nanocatalysis (CAN-USTC), University of Science and Technology of China, Hefei 230026 China; 2 State Key Laboratory of Chemical Resource Engineering, Beijing University of Chemical Technology, Beijing 100029 China; 3 Hefei National Laboratory for Physical Sciences at the Microscale, University of Science and Technology of China, Hefei 230026 China; 4 Department of Chemistry, Tsinghua University, Beijing 100084 China; 5 Collaborative Innovation Center for Nanomaterial Science and Engineering, Tsinghua University, Beijing 100084 China

## Abstract

The low activity of the oxygen reduction reaction in polymer electrolyte membrane fuel cells is a major barrier for electrocatalysis, and hence needs to be optimized. Tuning the surface electronic structure of platinum-based bimetallic alloys, a promising oxygen reduction reaction catalyst, plays a key role in controlling its interaction with reactants, and thus affects the efficiency. Here we report that a dealloying process can be utilized to experimentally fabricate the interface between dealloyed platinum–nickel alloy and amorphous nickel boride membrane. The coating membrane works as an electron acceptor to tune the surface electronic structure of the platinum–nickel catalyst, and this composite catalyst composed of crystalline platinum–nickel covered by amorphous nickel boride achieves a 27-times enhancement in mass activity relative to commercial platinum/carbon at 0.9 V for the oxygen reduction reaction performance. Moreover, this interactional effect between a crystalline surface and amorphous membrane can be readily generalized to facilitate the 3-times higher catalytic activity of commercial platinum/carbon.

## Abstract (web summary)

Low activity of the oxygen reduction reaction (ORR) in polymer electrolyte membrane fuel cells is a barrier for commercial applications. Here the authors report a nickel boride membrane-coated Pt-Ni catalyst, achieving 27-fold enhancement in mass activity for ORR relative to commercial Pt/C.

## Graphical abstract

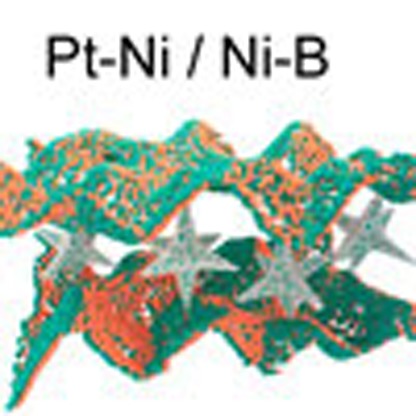

Pt-based bimetallic catalysts such as Pt–Co, Pt–Ni, Pt–Fe and so on are one type of the most efficient and important cathodic electrocatalysts for polymer electrolyte fuel cells due to their high activity towards the oxygen reduction reaction (ORR)^1^,^2^. Several crucial parameters such as crystal face^3^, d-band centre^4^, lattice strain^5^ and surface segregation^6^,^7^, which are related to their inherent electronic and geometric structures, have been posited to explain the performance of Pt-based bimetallic catalysts. In addition to tailoring the intrinsic structure of the Pt-based bimetallic catalyst itself, some other external factors involving the support^8^, ligands^9^,^10^,^11^, hybrid structures^12^ that enable the electron donor–acceptor effect between metal and its counterpart also play a vital role in improving catalytic activity, selectivity and stability. Driven by the need to find catalysts with optimized catalytic performance for practical applications, substantial efforts have been made to build composite catalysts with interfaces such as metal–metal^13^, metal–oxide^14^, metal–organic^15^ interface and so on.

With its unique short-range ordered and long-ranged disordered atomic alignment, amorphous alloys derived from crystalline material exhibit relatively unique physical and chemical properties due to their high concentration of coordinatively unsaturated sites, tunable compositions and plastic-like structures^16^. As one of the most promising catalysts, amorphous metal-boron alloy displays a wide range of applications in petrochemical production, energy conversion and environmental catalysis^17^. In material science, the bonding structure of amorphous metal-boron is intrinsically different from its crystalline counterparts because of the covalent component in its metal–metalloid bonds^18^, which would result in an obstacle for constructing interfaces between the crystalline metal and the amorphous metal-boron alloy. The effective synthetic methodology of integrating the crystalline material with amorphous metal-boron alloy and investigations of their synergetic effect are desired, yet challenging.

Here we present a chemical etching process that exploits the structural evolution of PtNi_3_ alloy in sodium borohydride solution; specifically, octahedral PtNi_3_ nanoparticles (NPs) are converted to concave octahedrons with surfaces encapsulated by an amorphous Ni–B membrane. This modified chemical etching strategy opens up a synthetic route to transform Pt–M (M=Ni, Co, Fe and so on) alloy to Pt–M/M–B hybrid structures by combining the chemical etching process and growth of M–B membrane. The close interaction and interfaces between Pt–Ni alloy and amorphous Ni–B alloy plays a positive role in enhancing the catalytic activity for the ORR. Surprisingly, this synergetic effect between crystalline metal and amorphous metal can be generalized to further facilitate the catalytic performance of commercial Pt/C, which is the most commonly used catalyst for practical applications.

## Results

### Synthesis and characterization

**Figure 1:**
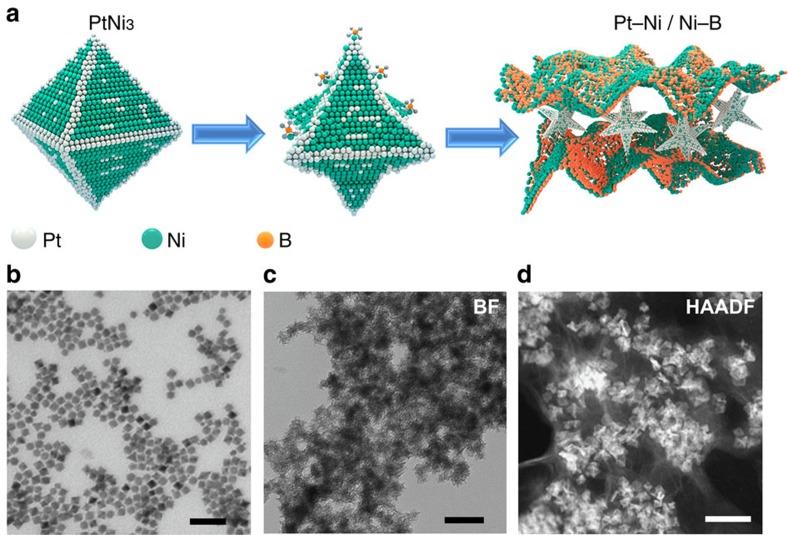
Scheme and TEM images of Ni–B membrane growing process. (**a**) Scheme illustrating the structural transformation from PtNi_3_ octahedron to Pt-Ni/Ni–B composite. TEM images of (**b**) initial PtNi_3_ octahedrons and (**c**) Pt-Ni/Ni–B composite. (**d**) High-angle annular dark-field scanning transmission electron microscope (HAADF-STEM) image of Pt-Ni/Ni–B composite. Scale bars, 50 nm.

The scheme summarizing the structural transformation of Pt–Ni alloy to Pt–Ni-based composite is illustrated in Fig. 1a. Figure 1b shows the transmission electron microscopy (TEM) image of PtNi_3_ octahedrons (12.5±1.5 nm) prepared by our previously reported method^19^. The hydrophilic surfactant of poly(vinylpyrrolidone) (PVP) ensured the favourable interaction of these monodispersed NPs with polar solvents, which is benefitial for the subsequent systematic investigation in aqueous solution. The parent PtNi_3_ polyhedrons were first dispersed in water and kept stirring for 5 min, followed by introducing a fresh sodium borohydride solution. After stirring for another 30 min, the as-prepared products were collected after washing and centrifugation. It was speculated that Pt–Ni alloy would undergo a chemical etching process, which was induced by the active BH_4_^−^. Figure 1c,d is the TEM and high-angle annular dark-field scanning transmission electron microscope images of the as-obtained hybrid Pt–Ni/Ni–B structures, respectively. The dealloyed Pt–Ni NPs were covered by an atomically thick membrane with lower contrast, accompanied by the transformation of the Pt–Ni octahedrons to concave octahedrons.

**Figure 2:**
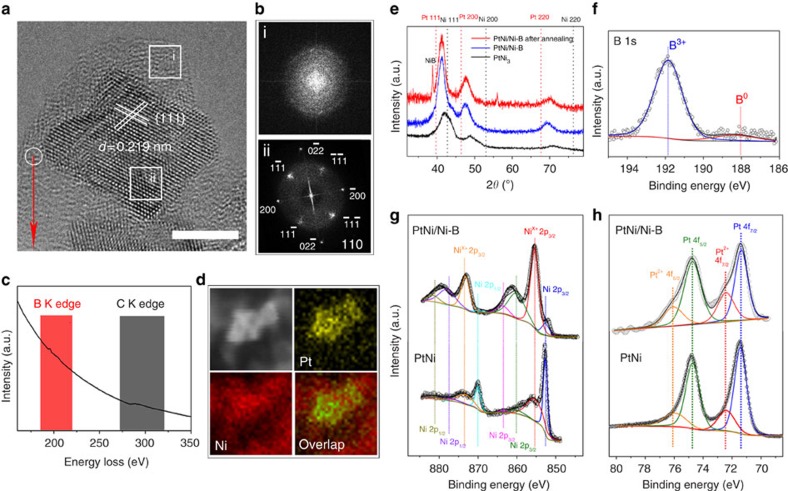
Characterization of PtNi/Ni–B composite. (**a**) High-angle annular dark-field scanning transmission electron microscope image. (**b**) Fast Fourier transform images correspond to the selected areas. (**c**) Electron energy loss spectroscopy measurement corresponds to the selected area. (**d**) Energy-dispersive X-ray elemental mapping results. (**e**) X-ray diffraction patterns of PtNi_3_ octahedrons, PtNi/Ni–B composite before and after annealing at 450 °C. X-ray photoelectron spectroscopy spectrum of (**f**) B 1*s*, (**g**) Ni 2*p* and (**h**) Pt 4*f*. Scale bars, 5 nm.

Spherical aberration-corrected high-resolution TEM (SA-corrected HRTEM) measurements were conducted to elucidate the microstructures of the hybrid Pt–Ni/Ni–B NPs. As shown in Fig. 2a, the amorphous Ni–B is characterized by long-ranged disordered atomic alignment, and the dealloyed Pt–Ni alloy shows the geometry and symmetry of concave octahedrons. The atomic picture presents a high density of atomic steps and defects on the {111} surface of obtained concave octahedral NPs. The fast Fourier transform image belonging to the Ni–B amorphous membrane does not show any clear diffraction spots, indicating the poor crystallinity of the membrane. Meanwhile, the fast Fourier transform patterns corresponding to the dealloyed Pt–Ni NPs along the direction demonstrate that the resulting NPs retain periodic structure and good crystallinity, reinforcing the nature of hybrid structures including the interface of amorphous and crystalline alloys (Fig. 2b). These membrane structures were sensitive to electron beam irradiation in SA-corrected HRTEM, which would result in increased crystallinity with exposure time (Supplementary Fig. 1). Further characterization using electron energy loss spectroscopy detected an apparent signal ascribed to the k-edge of B, which proves the existence of elemental boron in the membrane (Fig. 2c). The energy-dispersive X-ray elemental mappings of this hybrid structure confirm that the dealloyed NPs are composed of Ni and Pt (Fig. 2d). The homogeneous distribution of nickel throughout the whole membrane revealed that nickel is a major element in the obtained amorphous membrane. These results imply that Ni can be etched out from the PtNi_3_ alloy and react with borohydride ions to form a Ni–B membrane, which covers the dealloyed NPs.

It is worth mentioning that traditional dealloying process usually involve an oxidative etching process in which zero-valence metal atoms are oxidized to corresponding ions in the presence of an oxidant such as O_2_, H^+^, halides and so on^20^. Since borohydrides are usually used as a strong reducing agent in the synthesis of metals, this is not favourable for the oxidation of metal even under air exposure in the presence of borohydrides^21^. A control etching experiment with the same conditions except under nitrogen protection also enabled the formation of this hybrid ‘crystal-amorphous' structure, verifying that O_2_ is not a prerequisite in this chemical etching process (Supplementary Fig. 2). Hence, the essential process of chemical etching reported in this work is believed to be different from the traditional oxidative etching. We speculate that the actual process is dependent on the following three equations:


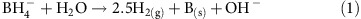



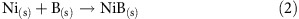



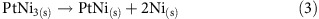


First, the Ni(0) in PtNi_3_ may serve as a catalyst to enhance the decomposition of the borohydride tetrahedron into solid B_(s)_ (ref. 22) (equation 1). The generated B_(s)_ then undergoes an alloying process immediately with the active Ni(0) of the PtNi_3_ alloy according to equation (2) to form an amorphous Ni–B alloy membrane *in situ* on the surface of the dealloyed Pt–Ni NPs. Simultaneously, the Ni dissolves from the PtNi_3_ alloy, and the remaining Pt and Ni rearrange and transform into a concave octahedral structure^23^. The processes following equations (1) and (3) are both accelerated by the formation of Ni–B membrane, which is assigned to equation (2). As such, the dealloying process and growth of Ni–B are linked and cooperatively promote one another. The chemical etching strategy reported here for the design of interfaces between bimetallic Pt–M crystalline alloy and amorphous M–B membrane may be readily generalized to other bimetallic alloys such as Pt–Co and Pt–Fe (Supplementary Fig. 3). Furthermore, density functional theory calculations showed that the B atom has superior adhesion to Pt(111) compared with the Ni atom. The binding energy difference (∼2.8 eV), and different sources of B and Ni species in the synthetic strategy both indicate that the nucleation/growth of Ni–B membrane starts from the B anchored on the NPs. This supports our above speculation for the actual process. This preferential growth enables the B–Pt binding to be substantial in the composite.

X-ray diffraction and inductively coupled plasma-mass spectrometry (ICP-MS) were used to elucidate the phase evolution and bulk composition changes occurring during the chemical etching process (Fig. 2e, Supplementary Fig. 4 and Supplementary Table 1). The pattern of the initial PtNi_3_ polyhedrons shows three major peaks, located between the Pt and Ni standard peaks, assigned to typical (111), (200) and (220) facets of the face-centred cubic phase. A slight shift towards lower 2*θ* value was observed after the chemical etching process, suggesting increased lattice spacing resulting from dissolution of Ni from the Ni-rich alloys. Meanwhile, no other evident peaks corresponding to the crystalline Ni–B or Ni(BO_2_)_2_ emerged, demonstrating that the generated membrane is poorly crystallized and further hydrolysis of borohydride has been effectively avoided^24^,^25^. To enhance the crystallinity of amorphous membrane, we further annealed the as-obtained composite at 450 °C under the protection of nitrogen. Peaks belonging to crystalline nickel boride appeared, which match the results studied by SA-corrected HRTEM that some sintering and crystalline Ni–B NPs were observed in the sample of annealed Pt–Ni/Ni–B composite (Supplementary Fig. 5)^26^. Noteworthy is the fact that the covering membrane of Ni–B was stable after being immersed in 0.1 M HClO_4_ solution for 24 h, showing high corrosion resistance (Supplementary Fig. 6). Furthermore, this membrane endowed a confinement effect that prevents dealloyed Pt–Ni NPs from aggregation at high temperatures, which is supported by the constant half peak width of the Pt–Ni alloy. All of these findings suggest the close relationship between the growth of amorphous Ni–B membrane and the chemical etching of the Pt–Ni alloy.

X-ray photoelectron spectroscopy was performed to identify the valence state of PtNi/Ni–B composite and electron transfer between PtNi and Ni–B membrane (Supplementary Fig. 4). As shown in Fig. 2f, the B exists mainly in an oxidized state, which may result from the readily achieved oxidation process by the oxygen in air. In principle, the amorphous structure (Fig. 2a) allows the B in the membrane to be often coordinatively unsaturated even after binding with Pt/Ni, and therefore highly reactive. More typically, the boron oxide (for example, BO_2_) is theoretically expected to be present because the anchored B has a strong attraction for the O on or close to Pt(111) for coordinative saturation (Supplementary Fig. 7). A similar case can be found in nickel boride catalysts^27^. Unlike the traditional case of metal boride, this oxidized B lacks electrons due to the unoccupied *p* orbitals and acts as a Lewis acid. For the case of PtNi/Ni–B, the Ni 2*p* core-level binding energy was clearly upshifted and the signal ascribed to Ni^2+^ increased. Meanwhile, the peaks belonging to Pt^2+^ 4*f* slightly increased with respect to the PtNi alloy (Fig. 2g,h). These results suggest effective electron transfer from PtNi alloy to the Ni–B membrane. In addition, Bader charge analysis indicates that 1.05 e are transferred from B to O in BO_2_, which is consistent with B^*δ+*^ by the X-ray photoelectron spectroscopy measurement (Fig. 2f); and simultaneously 0.59 e is transferred from Pt(111) to BO_2_ species, an indication of the electron-deficient nature (Fig. 2h). All of these findings support the conclusion that oxidation of Ni–B membrane is spontaneous in our synthesis. That is to say, it is the boron oxides (BO_*x*_) and not the B atoms in the B–Ni membrane that influence the electronic properties of the Pt surface by its interactions (that is, electron transfer), like as the active phase-support interaction of supported catalysts^28^.

### Electrochemical properties

**Figure 3:**
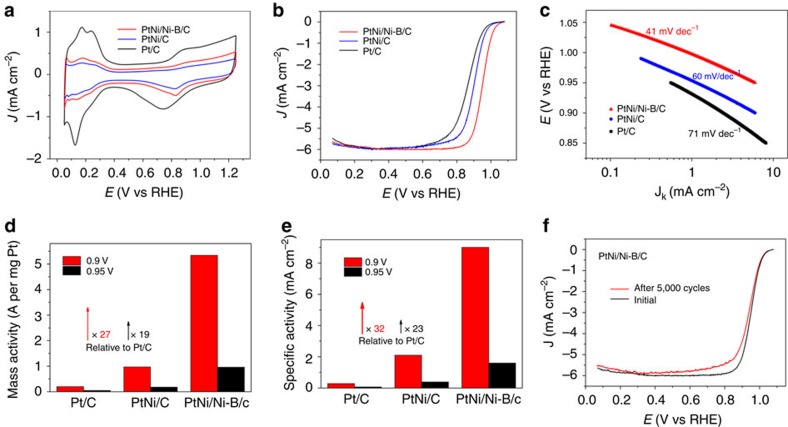
Electrochemical properties of PtNi/Ni–B. (**a**) Cyclic voltammetry curves of PtNi/Ni–B/C, PtNi/C and Pt/C catalyst in N_2_-saturated 0.1 M HClO_4_ solution with a sweep rate of 50 mV s^−1^. (**b**) ORR polarization curves. (**c**) The corresponding Tafel plots for PtNi/Ni–B/C, PtNi/C and Pt/C in O_2_-saturated 0.1 M HClO_4_ solution with a sweep rate of 10 mV s^−1^ and a rotation rate of 1,600 r.p.m. The currents were normalized to the geometric area of the rotating disk electrode (0.196 cm^2^). (**d**) Mass activity for these three catalysts at 0.9 and 0.95 V versus RHE. For the PtNi/C or PtNi/Ni–B/C catalyst, the Pt loading on a RDE was 1.2 μg, whereas the Pt loading was 2.4 μg for the Pt/C catalyst. (**e**) Comparison of specific activity at 0.9 and 0.95 V based on the ECSAs calculated from the charges associated with H_upd_. (**f**) ORR polarization curves of PtNi/Ni–B/C before and after 5,000 cycles between 0.6 and 1.0 V.

The electrocatalytic performance of PtNi/Ni–B/C towards ORR was evaluated and compared with as-prepared PtNi/C octahedrons (Supplementary Fig. 8) and commercial Pt/C catalyst (Johnson Matthey) (20% by wt of ∼3 nm Pt NPs on Vulcan XC-72 carbon support). Figure 3a shows the cyclic voltammetry curves of these three catalysts recorded at room temperature in N_2_-sutated 0.1 M HClO_4_ solution with a sweep rate of 50 mV s^−1^. The peaks at the potential range between 0.05<*E*<0.35 V on the cyclic voltammetry curves correspond to H_upd_ adsorption/desorption processes (H^+^+e^−^=H_upd_). The electrochemically active surface area (ECSA) can be obtained by calculating H_upd_ adsorption/desorption peak areas after double-layer correction (ECSA_Hupd_), assuming a value of 210 μC cm^−2^ for the adsorption in a hydrogen monolayer^29^. The specific ECSA_Hupd_ of Pt/C (71 m^2^ per g Pt) was higher than that of Pt–Ni catalysts due to a relatively small overall particle size. Meanwhile, the PtNi/Ni–B/C shows an increased ECSA_Hupd_ (59 m^2^ per g Pt) relative to that of PtNi/C (46 m^2^ per g Pt), indicating an increased catalytic surface after the etching process. The flexible amorphous Ni–B membrane did not affect the contact of reactants with Pt–Ni surface.

Figure 3b shows positive-going ORR polarization curves in the following order: Pt/C<PtNi/C<PtNi/Ni-B/C. A significant increase in ORR activity for PtNi/Ni–B/C was observed relative to Pt/C, along with Tafel slopes from 71 to 41 mV dec^−1^ (Fig. 3c). This value is in agreement with that of Pt_3_Ni-skin and Pt_3_Ni nanoframes reported recently^30^,^31^, but much lower than those commonly reported for Pt-based catalysts^32^. The kinetic current was calculated from the ORR polarization curve by the Koutecky–Levich equation and normalized to the loading amount of metal to compare the mass activity (Fig. 3d). The PtNi/Ni–B/C exhibited a mass activity of 5.3 A per mg Pt at 0.9 V versus reversible hydrogen electrode (RHE), which was ∼27 times higher than that of the commercial Pt/C catalyst (0.2 A mg^−1^). A similar enhancement (19-fold) was observed at 0.95 V versus RHE, which was chosen to minimize diffusion-induced errors on the calculation of kinetic currents.

The intrinsic activities of these catalysts were then evaluated by normalizing the mass activities to the ECSA in H_upd_ peaks. The PtNi/Ni–B/C had a specific activity of 9.05 mA cm^−2^ (specific activities per ECSA_Hupd_ of Pt) which was 32 times higher than that of Pt/C (0.28 mA cm^−2^) and 4 times higher than that of PtNi/C catalyst at 0.9 V (Fig. 3e). A similar enhanced trend (23-fold and 4-fold) was also observed at 0.95 V. Indeed, the as-prepared Pt–Ni octahedron can be modified by directly depositing a Ni–B membrane on its surface, thereby forming a PtNi/C/Ni–B composite. The ORR activity of PtNi/C/Ni–B showed 2.3 times higher of mass activity at 0.95 V than that of bare PtNi/C, however, a little lower than that of PtNi/Ni–B/C catalyst obtained by the *in situ* strategy (Supplementary Fig 9 and Supplementary Tables 2 and 3). Thus, the enhancement of the ORR activity of the PtNi/Ni–B/C, relative to the PtNi/C/Ni–B, could be attributed to the close interaction and interfaces between Pt–Ni alloy and amorphous Ni–B alloy, which would allow the pronounced electron transfer to occur, and the positive modulation of electron structures of Pt surfaces to be possible. The stability test of PtNi/Ni–B/C during potential cycling was performed using a linear potential sweeps between 0.6 and 1.0 V at 50 mV s^−1^ in an O_2_-saturated 0.1 M HClO_4_ solution (Fig. 3f).

**Figure 4:**
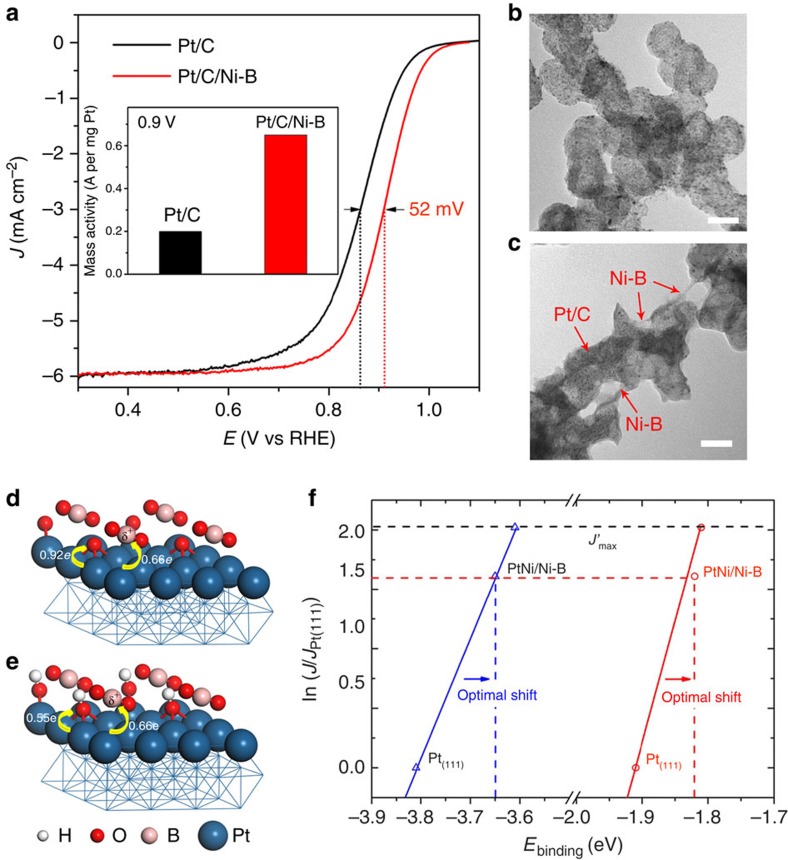
Electrochemical properties of Pt/C/Ni–B and density functional theory analysis. (**a**) ORR polarization curves and (inset) mass activities of Pt/C and Pt/C/Ni–B at 0.9 V versus RHE. (**b**,**c**) TEM images of Pt/C (**b**) and Pt/C/Ni–B (**c**). (**d**) Adsorption of O on the hollow site of BO_2_/Pt(111), including valence state of B and electron transfer from Pt(111) to BO_2_ and O. (**e**) Adsorption of OH on the hollow site of BO_2_/Pt(111), including valence state of B and electron transfer from Pt(111) to BO_2_ and OH. (**f**) The experimental current density as a function of the calculated binding energy of O (blue) and OH (red), showing optimal shift in binding energy for the maximum ORR activity by the arrow. The nominal maximum *J′*_max_ with respect to Pt(111) is deduced from the activity volcano for ORR proposed by Nørskov *et al*. Scale bars, 50 nm.

We also investigated this synergetic effect between commercial Pt/C catalyst and amorphous Ni–B membrane for ORR performance (Fig. 4a and Supplementary Fig. 10). Figure 4b,c is the TEM images of the Pt/C catalyst before and after surface modification of Ni–B. Clearly, the Pt/C was covered by an atomically thick membrane with lower contrast, which is similar to the PtNi/Ni–B. As a result, the Pt/C/Ni–B shows an ∼52 mV positive shift in half-wave potential (*E*_1/2_), indicating the improved activity (threefold mass activity at 0.9 V) over Pt/C (Fig. 4a). Moreover, the Pt/C/Ni–B shows an enhanced durability during a 5,000 cycle stability test compared with Pt/C catalyst (Supplementary Fig. 11), which may result from the Ni–B membrane effectively preventing the agglomeration of Pt NPs. According to the X-ray diffraction patterns, no peak assigned to Pt–Ni alloy was observed after this surface modification (Supplementary Fig. 12), which strongly suggests the enhancement of activity and stability are not attributable to the alloying process of Pt/C with Ni.

## Discussion

Density functional theory calculations were carried out to elucidate, at the microscopic level, the origin of the enhanced performance of composite catalysts in ORR by use of the binding energies of O and OH. As demonstrated by our samples, boron oxides are stably present in the amorphous membrane due to spontaneous coordinative saturation of the B and strong attraction to O. In what follows, we take BO_2_ as an example (its rationality and reliability can be seen in the method and model in Supplementary Note 1). According to the associative mechanism available to the ORR, Nørskov *et al*. provided a more reliable prediction for reactivity trends of metal/alloy catalysts using the binding energies of reaction intermediates (for example, O and OH) as a simple descriptor^33^,^34^,^35^. Our calculations show that BO_2_/Pt(111) has not only a weaker O binding energy (−3.65 eV) than that on Pt(111) (−3.82 eV) but also a lower OH binding energy by 0.09 eV with respect to Pt(111) (−1.82 versus −1.91 eV). As shown in Fig. 4d,e, the shifts in binding energies of the two descriptors on BO_2_/Pt (111) are well within the respective optimal ranges of better electrocatalyst for ORR proposed by Nørskov *et al*., that is, 0.2 eV for the O binding energy^36^ and 0.1 eV for the OH binding energy^37^, and the current density is expected to be close to the predicted maximum value (dashed line in Fig. 4f). This accords with the idea that the ideal catalyst for ORR needs to activate molecular oxygen while, at the same time, binding oxygen species such as O and OH only weakly. At this rate, by the simultaneous optimization of surface oxygen and oxygenated intermediate affinities of Pt (see the arrows in Fig. 4f), the right balance of O_2_ dissociation kinetics and O hydrogenation kinetics is most likely established on BO_2_/Pt(111), so the ORR rate is expected to be substantially enhanced over commercial Pt/C catalyst (Fig. 4a). In addition, the effects of subsurface Ni on the O binding in the ORR reaction are also explored from the geometrical (that is, lattice strain) and electronic (that is, alloying) perspectives, and it was found that the oxidation process of B by the oxygen and the optimal trend induced by BO_2_ remains unchanged (Supplementary Fig. 13 and Supplementary Note 1). Especially, with two Pt overlayers and a 1% compressive strain, the BO_2_/Pt-terminated PtNi surface binds O (−3.59 eV) closest to the optimal value. This means that in the hybrid system, the positive modulation of electron structure of reactive Pt surface is mainly dominated by the electron-deficient amorphous Ni–B alloy, rather than the Ni alloying effect. This result is consistent with our systematic experiments (PtNi/C/Ni–B composite versus PtNi/C versus PtNi/Ni-B/C; Supplementary Fig. 9 and Supplementary Table 2).

In total, we believe that in this composite, an electronic effect of Ni–B membrane on the Pt-O and Pt-OH chemical bonding is implemented by the electron-deficient boron oxide dominating electron transfer. The mechanism increasing surface site availability is different from that of Pt_3_Ni with Pt-skin^4^, but nonetheless also offers high ORR activity. Moreover, in practice, the amorphous membrane and ‘loose' interface (Fig. 2a), from the geometrical effects contribution, both ensure that available surface sites of internal Pt_3_Ni NPs are open for many reactants, similar to Pt_3_Ni nanoframes^6^. The strategy used here may pave the way for future development of efficient catalyst systems not only for the ORR but also for more practical reactions.

## Methods

### Reagents

Analytical grade benzyl alcohol, benzoic acid, sodium borohydride and nickel nitrate were obtained from Beijing Chemical Reagents, China. Pt(acac)_2_ (99%), Ni(acac)_2_ (99%) and PVP (molecular weight (MW)=8,000, analytic reagent (AR)) were purchased from Alfa Aesar. Nafion was acquired from Sigma-Aldrich. All of the chemicals used in this experiment were of analytical grade and used without further purification.

### Synthesis of PtNi_3_ and PtNi nanocrystal

A unit of 25 mg Pt(acac)_2_, 40 mg Ni(acac)_2_, 250 mg benzoic acid and 400 mg PVP (molecular weight=8,000) were dissolved 25 ml benzyl alcohol, followed by 15 min vigorous stirring. The resulting homogeneous green solution was transferred into a 50 ml Teflon-lined stainless-steel autoclave. The sealed vessel was then heated at 150 °C for a 12 h before it was cooled down to room temperature. The products were precipitated by acetone, separated via centrifugation and further purified by an ethanol–acetone mixture. The PtNi nanaocrystal was synthesized via similar procedures for the PtNi_3_ nanocrystal but with 15 mg Ni(acac)_2_ in the first step.

### Synthesis of PtNi/Ni–B

A unit of 2 mg Pt (based on ICP-MS measurement) as-prepared octahedron-shaped PtNi3 particles were dispersed in 5 ml deionized water and kept stirring for 5 min, followed by introducing 0.5 ml fresh sodium borohydride solution (20 mg solid borohydride in 1 ml deionized water). Kept stirring for 30 min, and the reaction was finished until no bubbles. Then the as-prepared products (PtNi/Ni–B) were collected after washing and centrifugation by deionized water.

### Synthesis of PtNi/Ni–B/C and PtNi/C

The carbon black-supported PtNi/Ni–B catalyst (PtNi/Ni–B/C) was prepared using the following procedures: 10 mg of carbon black (Vulcan XC-72) was first dispersed in 2.5 ml ethanol and 2.5 ml deionized water mixed solution. After 30 min vigorous stirring, 5 ml ethanol solution containing 2 mg Pt (base on ICP-MS measurement) as-prepared PtNi/Ni-B was added into the carbon suspensions with further ultrasonication for additional 30 min. Then the as-prepared products (PtNi/Ni–B/C) were collected after washing and centrifugation by ethanol and deionized water. The PtNi/C was synthesized by the similar procedures for the PtNi/Ni–B/C.

### Synthesis of Pt/C/Ni–B and PtNi/C/Ni–B

The amorphous Ni–B membrane modified commercial Pt/C catalyst (Johnson Matthey Corp., 20 wt% Pt on Vulcan XC-72) was prepared as follows, 5 mg Pt/C catalyst and 10 mg nickel nitrate were dispersed in 5 ml deionized water and kept stirring for 5 min, followed by introducing 0.5 ml fresh sodium borohydride solution (20 mg solid borohydride in 1 ml deionized water). Kept stirring for 30 min, and the reaction was finished until no bubbles. Then the as-prepared products (Pt/C/Ni–B) were collected after washing and centrifugation by ethanol. The PtNi/C/Ni–B was prepares by the similar procedures for the Pt/C/Ni–B.

### Electrochemical study

Electrochemical measurements were conducted at room temperature, with a three-electrode system controlled by a CHI760E electrochemical workstation (Chenhua Inc., Shanghai, China). A Pine AFMSRCE electrode rotator was used for rotating disk electrode (RDE) experiments. A silver/silver chloride (3 M KCl) electrode was used as the reference, and a platinum wire was used as the counter electrode. The electrolyte was a 0.1 M HClO_4_ aqueous solution. Prior to each measurement, the electrolyte was bubbled with oxygen or nitrogen until saturation. A constant O_2_ or N_2_ flow was then maintained in the headspace of the electrolyte during the whole experiment.

*Preparation of catalysts inks for RDE*. The synthesized octahedral PtNi nanocrystals, and PtNi/Ni-B were incorporated onto carbon black (Cabot, Vulcan XC-72), and washed by ethanol, anhydrous ether and water. Total metal loading was controlled in the range of ∼20%. Then the catalyst ink was prepared by ultrasonically 0.5 mg Pt (based on ICP-MS measurement) catalyst powder and 20 μl of 5 wt% Nafion solution were dispersed in 1 ml of an isopropanol and water mixture, followed by a sonication for at least 15 min to form a homogeneous catalyst ink. A volume of 3–5 μl of the catalyst inks were loaded onto a glassy carbon disk electrode with a diameter of 5 mm. The catalyst layer was allowed to dry under ambient conditions before an electrochemical measurement.

*Cyclic voltammetry on a static electrode*. Cyclic voltammetry measurements were carried out in 0.1 M HClO_4_ solutions under a flow of N_2_ at a sweep rate of 50 mV s^−1^. The electrochemically active surface area (ECSA) was estimated by measuring the charge associated with H_upd_ adsorption (*Q*_H_) between 0.05 and 0.37 V, and the specific ECSA was calculated based on the following relation^29^:


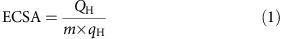


where *Q*_H_ is the charge for H_upd_ adsorption, *m* is the metal loading and *q*_H_ (210 μC cm^−2^) is the charge required for monolayer adsorption of hydrogen on Pt surfaces.

*RDE voltammetry*. To obtain steady state voltammetric data on an RDE, the catalyst ink was loaded on a glassy carbon RDE (5 mm diameter), which was then mounted on the electrode rotator. Potential of the catalyst-loaded RDE working electrode was scanned linearly and cathodically at a rate of 5 mV s^−1^ with a rotation speed at 1,600 r.p.m. For the kinetic current calculation, the Koutecky–Levich equation can be described as follows^38^:


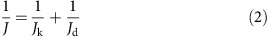


where *J* is the experimentally measured current density, *J*_k_ is the kinetic current density and *J*_d_ is the diffusion limiting current density, and the kinetic current density was calculated based on the following equation:


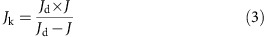


*Electrochemical accelerated durability tests*. Using the same system as in a cyclic voltammetry test, accelerated durability test was conducted in O_2_-saturated 0.1 M HClO_4_ solutions by potentials between 0.6 and 1.0 V versus RHE at a sweep rate of 50 mV s^−1^ for a given number of cycles.

### Date availability

The data that support the findings of this study are available from the corresponding authors on request.

## Additional information

**How to cite this article:** He, D. *et al*. Amorphous nickel boride membrane on a platinum–nickel alloy surface for enhanced oxygen reduction reaction. *Nat. Commun.* 7:12362 doi: 10.1038/ncomms12362 (2016).

## Supplementary Material

Supplementary Information Supplementary Figures 1-13, Supplementary Tables 1-3, Supplementary Note 1 and Supplementary References
