# Supplementary material for: Amorphous nickel boride membrane on a platinum–nickel alloy surface for enhanced oxygen reduction reaction
Source: Nat Commun. 2016 Aug 9;7:12362. doi: 10.1038/ncomms12362 (PMC4980487; doi:10.1038/ncomms12362)
Supplement: Supplementary Information — Supplementary Figures 1-13, Supplementary Tables 1-3, Supplementary Note 1 and Supplementary References [file ncomms12362-s1.pdf]

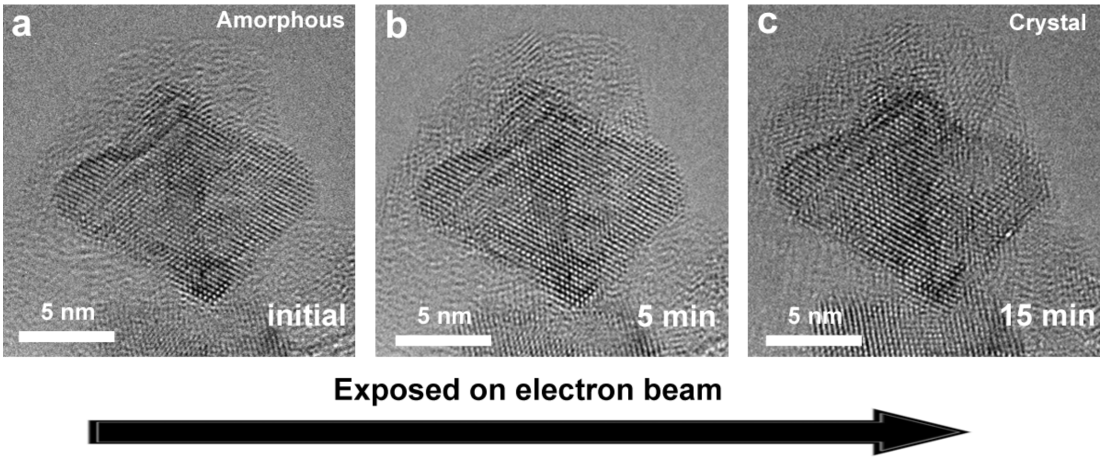

Supplementary Figure 1. HRTEM images of PtNi / Ni-B composite exposed to electron beam. The scale bars are 5 nm.

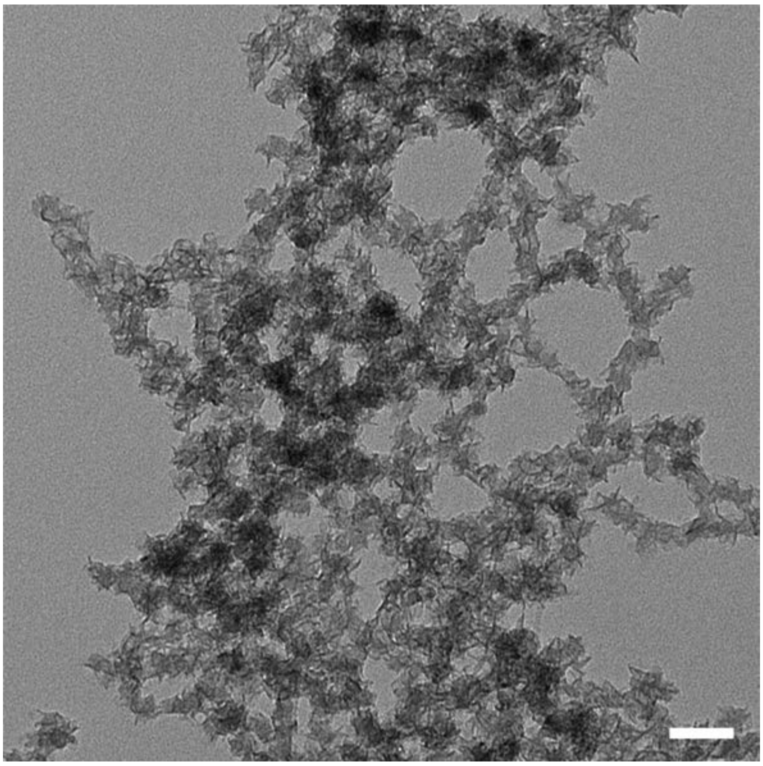

Supplementary Figure 2. TEM image of PtNi/Ni-B composite obtained under N_2 protection. The scale bars are 50 nm.

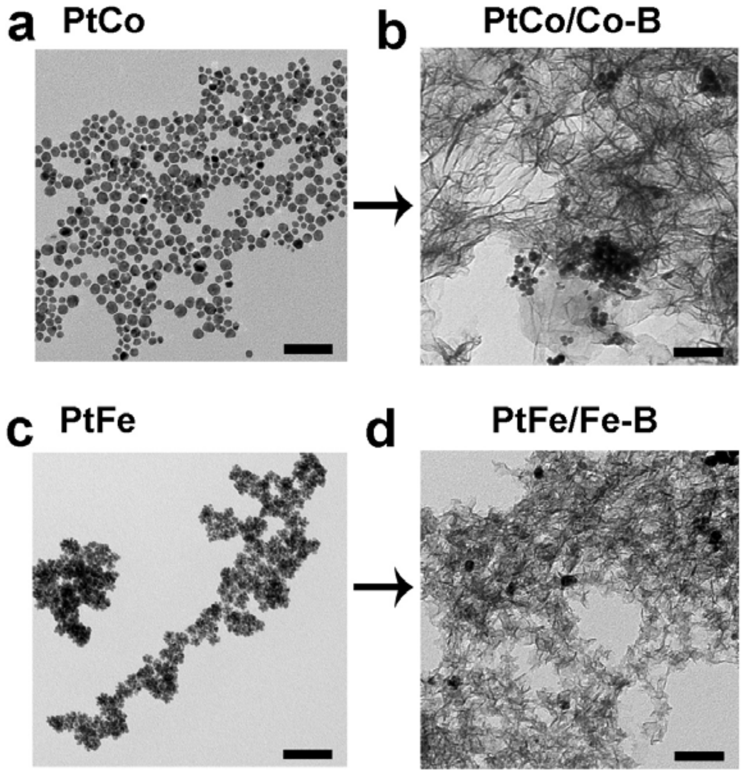

Supplementary Figure 3. The dealloying process and growth of M-B in PtCo (a, b) and PtFe (c, d) bimetallic systems. The scale bars are 50 nm.

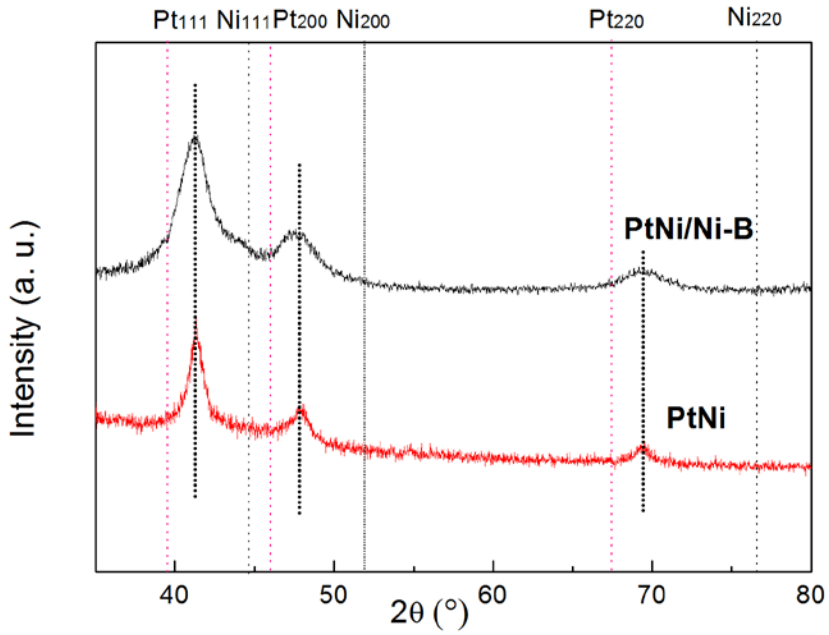

Supplementary Figure 4. XRD patterns of PtNi and PtNi/Ni-B.

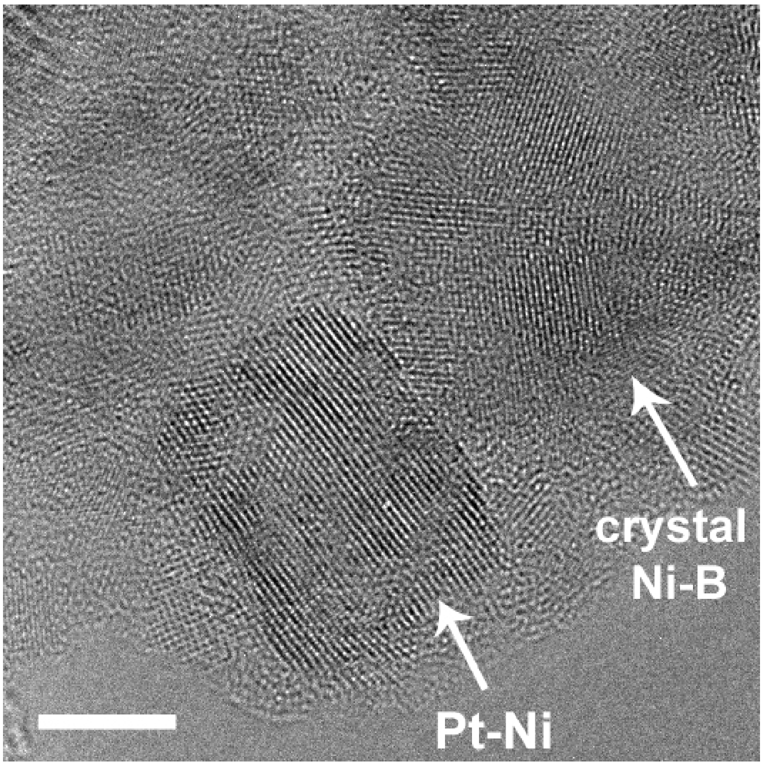

Supplementary Figure 5. HRTEM images of Pt-Ni / Ni-B composite after annealing at 450 ^{o}C in nitrogen flow. The scale bars are 5 nm.

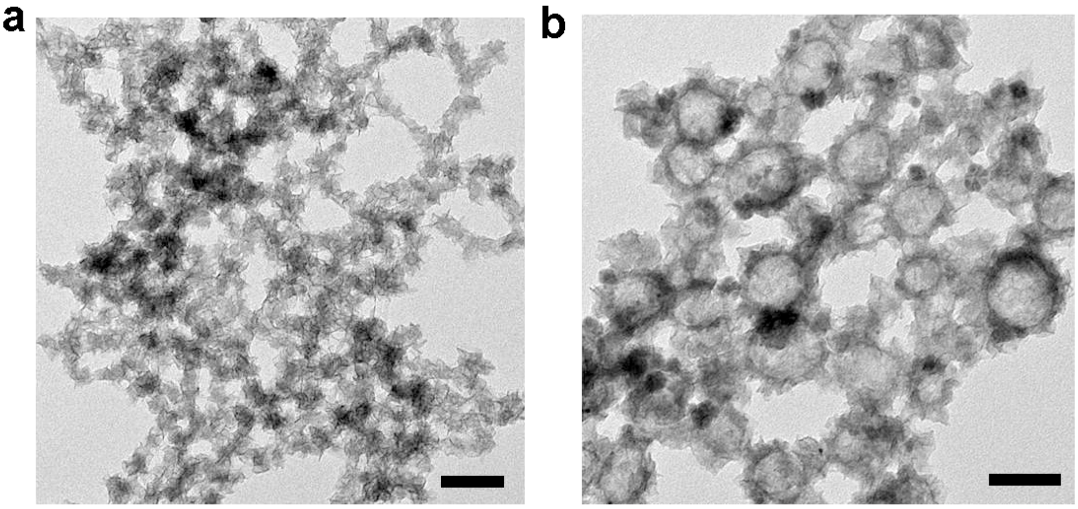

Supplementary Figure 6. TEM images of Pt-Ni / Ni-B composite after (A) dipping in 0.1 M $HClO_4$ solution for 48 h, and (B) annealing at 450 ^{o}C for 12 h in a nitrogen flow. The scale bars are 50 nm.

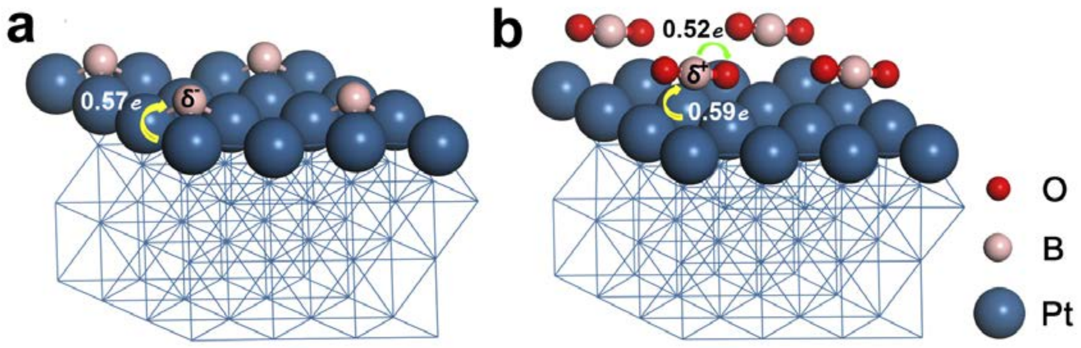

Supplementary Figure 7. (a**)** Adsorption of B on the hollow site of Pt(111), including valence state of B and electron transfer from Pt(111) to B. **(**b**)** Adsorption of BO_2 on the hollow site of Pt(111), including valence state of B and electron transfer from Pt(111) to BO_2.

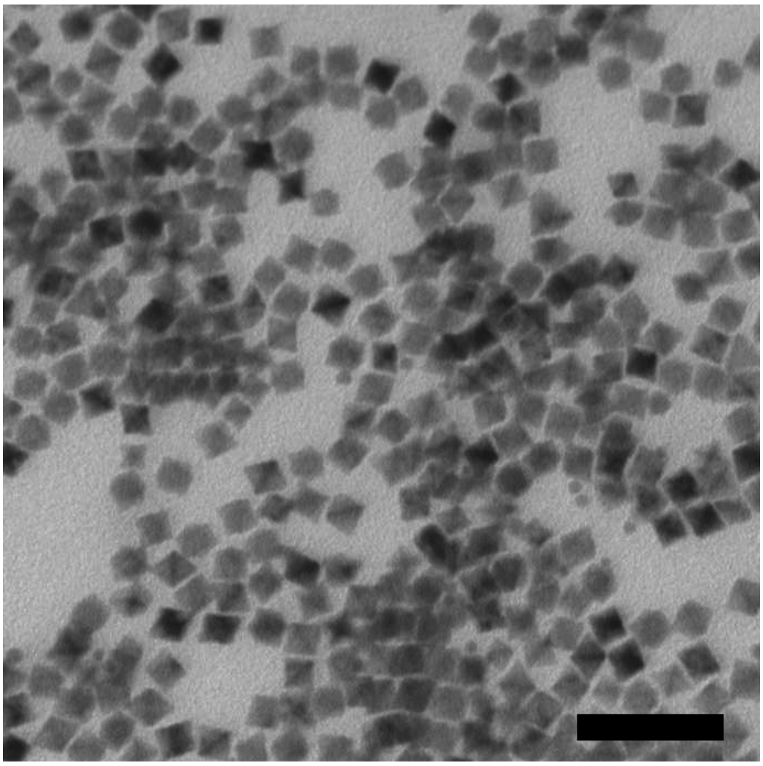

Supplementary Figure 8. TEM images of as-prepared PtNi octahedron. The scale bars are 50 nm.

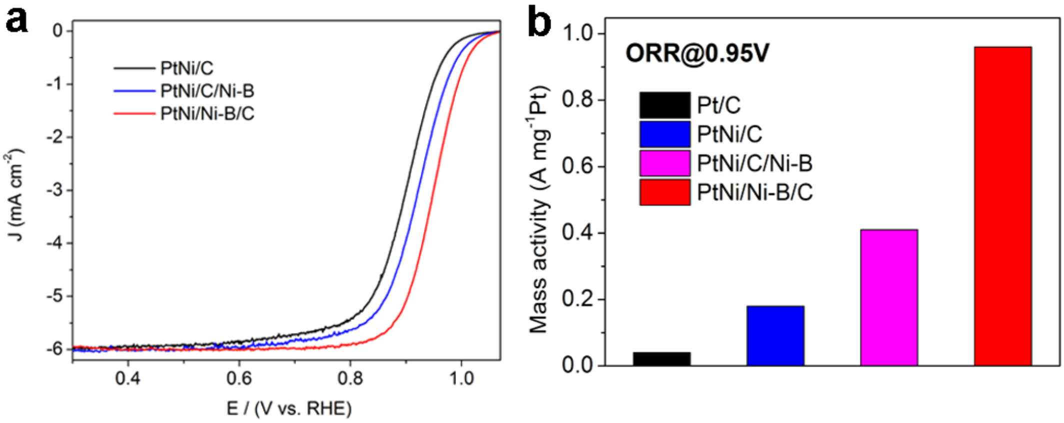

Supplementary Figure 9. (a) ORR polarization curves of PtNi/Ni-B/C, PtNi/C/Ni-B, Pt/Ni/C and Pt/C in O2-saturated 0.1M HClO4 solution with a sweep rate of 10 mV s-1 and a rotation rate of 1600 rpm. (b) Mass activity for these four catalysts at 0.95 V versus RHE.

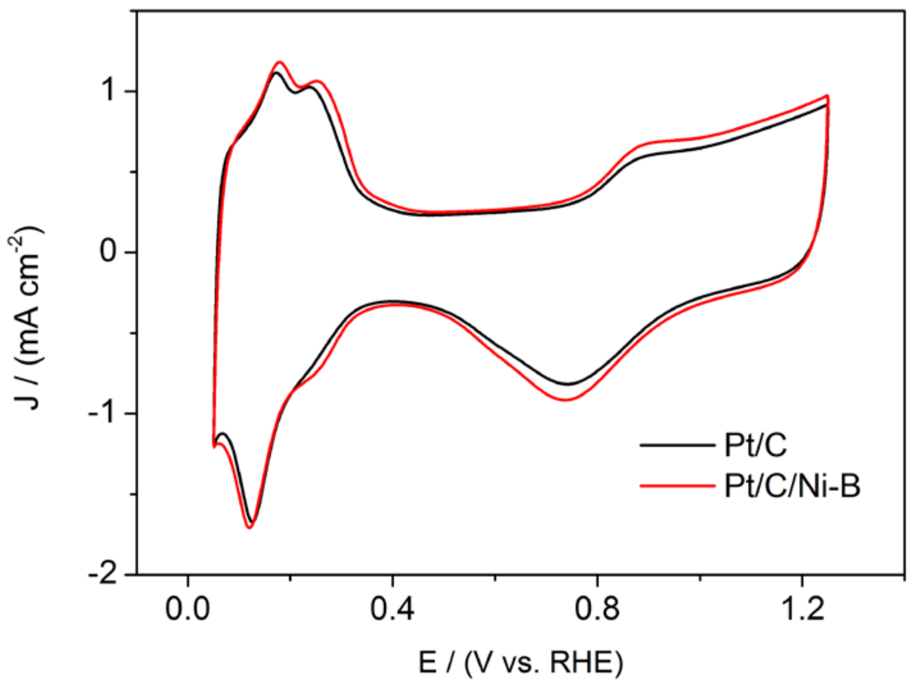

Supplementary Figure 10. CV curves of Pt/C and Pt/C/Ni-B in N_2-saturated 0.1M $HClO_4$ solution.

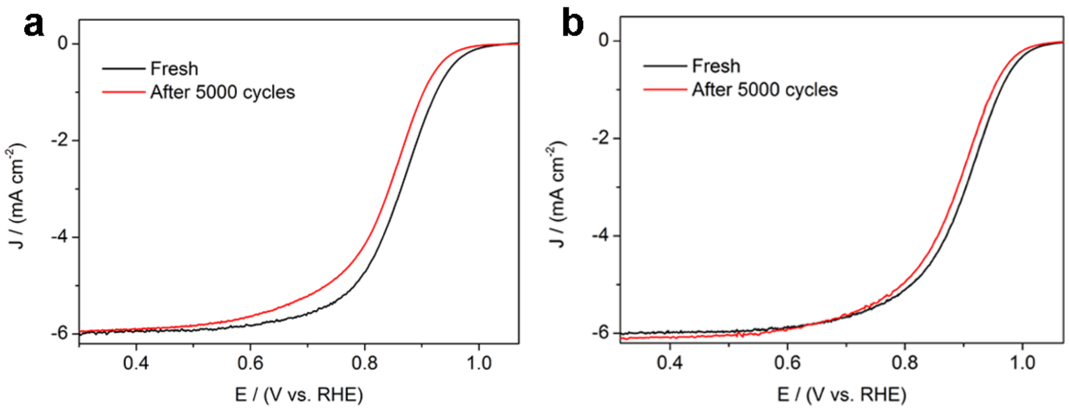

Supplementary Figure 11. ORR polarization curves of (A) Pt/C and (B) Pt/C/Ni-B before and after 5000 cycles between 0.6 and 1.0 V.

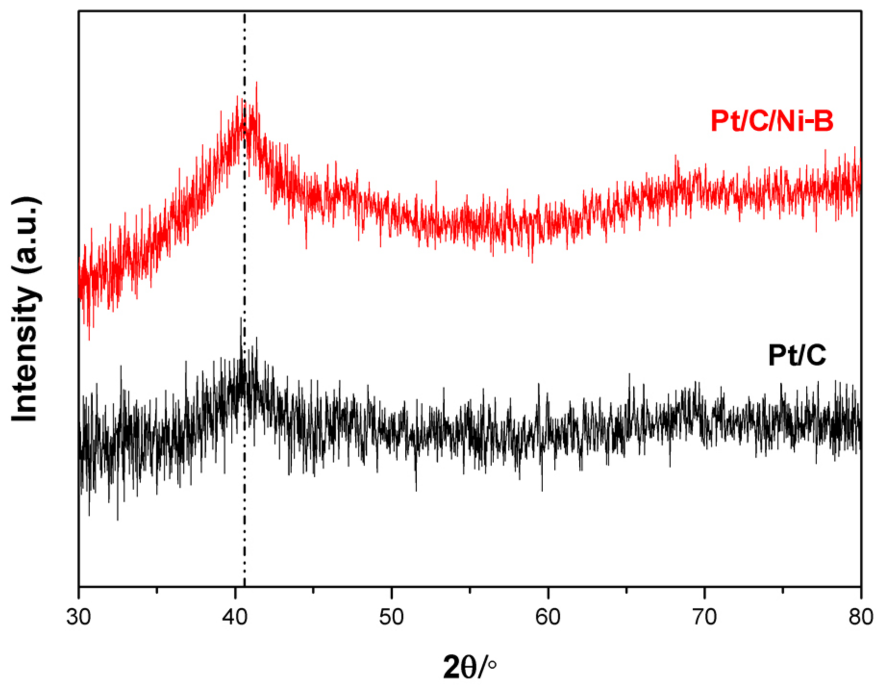

Supplementary Figure 12. XRD patterns of Pt/C and Pt/C/Ni-B.

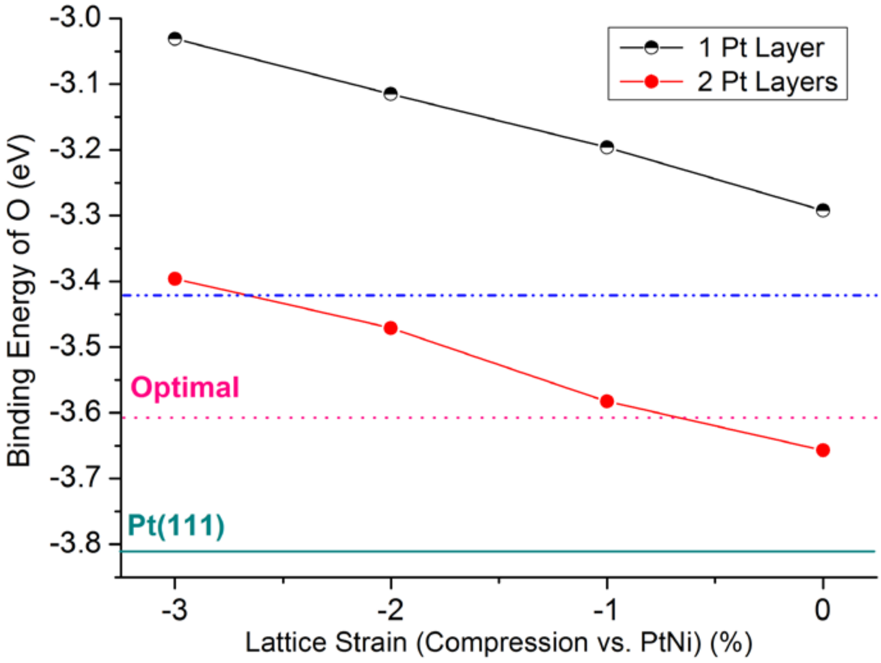

Supplementary Figure 13. Binding energy of O on Pt-terminated PtNi(111) surfaces as a function of compressive strain (with respect to bulk PtNi) and number of Pt overlayers. The pink dotted, and blue dashed-dotted horizontal lines represent the theoretically determined optimal binding energy of O and the lower limit of optimal range from the previous work[1], respectively. The green solid line shows the binding energy of O on Pt(111).

Supplementary Table 1. Atomic contents of Pt, Ni and B in PtNi/Ni-B, $PtNi_3$ and PtNi measured by ICP-MS.

Catalyst	Pt (μg mL^{-1})	Ni (μg mL^{-1})	B (μg mL^{-1})
PtNi/Ni-B	2.84	2.74	0.15
$PtNi_3$	2.53	2.16	
PtNi	2.93	0.77	

Supplementary Table 2. Surface areas and ORR activities at 0.9 V and 0.95 V versus RHE for PtNi/Ni-B/C, PtNi/C, Pt/C, Pt/C/Ni-B, PtNi/C/Ni-B catalysts.

Catalyst	Pt loading ($\mu g\ cm^{-2}$)	ECSA ($m^2\ g^{-1}$ Pt)	Mass activity at 0.9 V/0.95V ($A\ mg^{-1}$ Pt)	Specific activity at 0.9V/0.95V ($mA\ cm^{-2}$)
PtNi/Ni-B/C	6.1	59	5.34/0.96	9.05/1.62
PtNi/C	6.1	46	0.97/0.18	2.11/0.39
Pt/C	12.2	71	0.20/0.05	0.28/0.07
Pt/C/Ni-B	12.2	73	0.65/0.15	0.89/0.21
PtNi/C/Ni-B	6.1	47	1.87/0.41	3.98/0.87

Supplementary Table 3. Mass activities at 0.9, 0.92, 0.94 and 0.95 V versus RHE for PtNi/Ni-B/C, PtNi/C, Pt/Ccatalysts.

**Catalyst**	**0.9 V**	**0.92 V**	**0.94 V**	**0.95 V**
PtNi/Ni-B/C (A mg^{-1} Pt)	5.34	2.74	1.37	0.96
PtNi/C (A mg^{-1} Pt)	0.97	0.52	0.27	0.18
Pt/C (A mg^{-1} Pt)	0.20	0.11	0.06	0.05
[PtNi/Ni-B/C] : [Pt/C]	~27	~25	~23	~19

Supplementary Note 1

Methodology and calculation model All spin-polarized calculations were performed using a periodic plane-wave density functional theory (DFT) method, as implemented in the Vienna ab-initio simulation package (VASP)[2]. The RPBE exchange-correlation functional[3] of a generalized gradient approximation (GGA), and the projector augmented wave (PAW) method[4] for the electron-ion interaction were used. The kinetic energy cutoff was 408 eV. A four layer Pt(111) slab with a p (2 x 2) surface unit cell was adopted to modeled the extended Pt-Ni(111) reactive surface. The validity of the assumption is confirmed by previous experimental[5,6] and theoretical studies,4 which had unequivocally demonstrated that the Pt-Ni nanoalloys after chemical etching favor the metal surface segregation, i.e., Pt-rich (111) facets. The same assumption Pt(111) instead of $Pt_3Ni(111)$ was adopted in theoretical calculations, too[7]. The vacuum spacing in the direction normal to the surface was at least 12 Å between neighboring slab images. The Brillouin zone was sampled using a 6 x 6 x 1 Monkhorst-Pack k-point mesh[8]. Adsorbates and the top two layers of the slab were allowed to relax. The criterion of force convergence was set to 0.02 eV/Å.

The binding energy Eb, describing not only the membrane-NPs interaction but also the stability of oxygen, was defined as follows:

$$E_b = E_{total} - (E_{(composite)\ substrate} + E_{adsorbate})$$

where E_{total} is the total energy of the adsorbate-substrate system, $E_{(composite)\ substrate}$ and $E_{adsorbate}$ are the energies of the (pre-adsorbed or pure) substrate and the free adsorbate, respectively. The calculated binding energies of O and OH on Pt(111) (0.25 ML) were -3.82

and -1.91 eV, respectively, which are in good agreement with the previous studies (-3.81[9,10]) and 1.90 eV[11]). On the other hand, the much larger binding energy of B (-6.18 eV) compared with Ni (-3.38 eV) on Pt(111) at least indicates that the coated Ni-B membrane would allow contact with the internal Pt-Ni NPs mainly through the B-Pt bonds rather than the Ni-Pt ones. Aberration-corrected high-angle annular dark-field (HAADF) images shows the Ni-B membrane has an amorphous structure and does not completely cover Pt-Ni NPs. The two evidences indicate that the electronic properties of Pt surface are essentially controlled by the state that anchored B holds. To a certain extent, it also supports the hypothesis that B instead of B-Ni as a straightforward microscopic model. In the unique fashion, the use of model systems enables us to mimic the basic features of real composite catalysts, such as the membrane-NPs interaction and the change of valence of B. Bader charge analysis[12] showing charge transfer was employed to identify the valence states of various species present therein. The B on Pt(111) accepts 0.57 e due to different electronegativity, indicative of a negative valence of $B^{\delta-}$, which conflicts with the XPS measurement. This means the B in the Ni-B membrane does not stably exist only in the atomic/alloy states.

In addition, to further study the effects of subsurface Ni atoms on the O binding in the ORR reaction from the geometrical (i.e., lattice strain) and electronic (i.e., alloying) perspectives, a six layer PtNi(111) slab with the same p (2 x 2) surface unit cell was considered. In general, a compressive strain was induced in the surface Pt-skin of PtNi alloy because of the difference of lattice parameter between them. Therefore, in the O adsorption calculation of the complex system, we not only modeled the nanoparticles as PtNi(111) surfaces with 1 and 2 Pt overlayers for the practical Pt-skin after the etching, but also introduced the 1, 2 and 3% lateral compressive strain in the slab for the lattice mismatch. Results showed (Supplementary Figure 13) that the Pt-terminated surfaces of PtNi alloys bind oxygen

weaker than Pt(111), and the binding of O weakens monotonically with compressive strain. In detail, for the PtNi(111) with single Pt overlayer, all the O binding energies whether under compressive strain (1-3%) or not (0%), are far away from the lower limit of optimal range (-3.42 eV). While with two Pt overlayers, the uncompressed surface binds O (-3.65 eV) slightly stronger than the optimal one (-3.62 eV), and it is most close to the optimal value with a 1% compressive strain (-3.59 eV). Even if under 2% compressive strain, the O binding energy is in the optimal range. Considering that the surface Pt-skin of obtained concave octahedral NPs possesses numerous defects after the etching (Fig. 2A), we affirm that smaller lattice strain or compression (1-2%) is possible to occur.

Supplementary References

1 Stamenkovic, V. *et al.* Changing the activity of electrocatalysts for oxygen reduction by tuning the surface electronic structure. *Angewandte Chemie* **118**, 2963-2967 (2006).

2 Kresse, G. & Furthmüller, J. Efficient iterative schemes for ab initio total-energy calculations using a plane-wave basis set. *Phys Rev B* **54**, 11169-11186 (1996).

3 Hammer, B., Hansen, L. & Nørskov, B. J. K. Improved adsorption energetics within density-functional theory using revised Perdew-Burke-Ernzerhof functionals. *Phys Rev B* **59**, 7413-7421 (1999).

4 Blöchl, P. E. Projector augmented-wave method. *Phys Rev B* **50**, 17953-17979 (1994).

5 Wu, Y. *et al.* A strategy for designing a concave Pt–Ni alloy through controllable chemical etching. *Angewandte Chemie International Edition* **51**, 12524-12528 (2012).

6 Cui, C., Gan, L., Heggen, M., Rudi S., & Strasser P. Compositional segregation in shaped Pt alloy nanoparticles and their structural behaviour during electrocatalysis. *Nat. Mater.* **12**, 765–771 (2013).

7 Stamenkovic, V. R. *et al.*Improved oxygen reduction activity on Pt3Ni (111) via increased surface site availability. *Science* **315**, 493-497 (2007).

8 Monkhorst, H. J. & Pack, J. D. Special points for Brillouin-zone integrations. *Phys Rev B* **13**, 5188-5192(1976).

9 Herron, J. A. *et al.* Oxygen reduction reaction on platinum-terminated “onion-structured” alloy catalysts. *Electrocatalysis* **3**, 192-202 (2012).

10 Wang, G. *et al.* Pt Skin on AuCu Intermetallic Substrate: A Strategy to Maximize Pt Utilization for Fuel Cells. *J Am Chem Soc* **136**, 9643-9649 (2014).

11 Jinnouchi, R., Kodama, K. & Morimoto, Y. DFT calculations on H, OH and O adsorbate formations on Pt (111) and Pt (332) electrodes. *J Electroanal Chem* **716**, 31-44 (2014).

12 Henkelman, G., Arnaldsson, A. & Jónsson, H. A fast and robust algorithm for Bader decomposition of charge density. *Comp Mater Sci* **36**, 354-360 (2006).
